# Retraction: Naringenin inhibits migration, invasion, induces apoptosis in human lung cancer cells and arrests tumour progression in vitro

**DOI:** 10.1111/jcmm.18325

**Published:** 2024-04-29

## Abstract

Shi X, Luo X, Chen T, Guo W, Liang C, Tang S, Mo J. Naringenin inhibits migration, invasion, induces apoptosis in human lung cancer cells and arrests tumour progression in vitro. *J Cell Mol Med*. 2021; 25: 2563–2571. https://doi.org/10.1111/jcmm.16226

The above article, published online on 1 February 2021 in Wiley Online Library (wileyonlinelibrary.com), has been retracted by agreement between the journal Editor‐in‐Chief, Stefan N. Constantinescu, and the Foundation for Cellular and Molecular Medicine/John Wiley & Sons Ltd. Following publication, concerns were raised by third parties that portions of Figure 1 are identical to Figure 1 in another article (1) from a different author group and covering a different research topic. Further investigation confirmed these concerns and revealed additional duplication in Figures 1 and 3, overlapping with Figures 1 and 2 in (1). The authors of both articles were approached for an explanation but were unable to address the concerns raised. The authors of (1) did not respond, and the authors of this article were unable to provide any underlying data. The retraction has been agreed because of concerns regarding the integrity of the research presented given that the figures were duplicated.

(1) Zhang J, Wang R, Cheng L, Xu H. Celastrol inhibit the proliferation, invasion and migration of human cervical HeLa cancer cells through down‐regulation of MMP‐2 and MMP‐9. *J Cell Mol Med*. 2021; 25: 5335‐5338. https://doi.org/10.1111/jcmm.16488

Shi X, Luo X, Chen T, Guo W, Liang C, Tang S, Mo J. Naringenin inhibits migration, invasion, induces apoptosis in human lung cancer cells and arrests tumour progression in vitro. *J Cell Mol Med*. 2021; 25: 2563–2571. https://doi.org/10.1111/jcmm.16226

The above article, published online on 1 February 2021 in Wiley Online Library (wileyonlinelibrary.com), has been retracted by agreement between the journal Editor‐in‐Chief, Stefan N. Constantinescu, and the Foundation for Cellular and Molecular Medicine/John Wiley & Sons Ltd. Following publication, concerns were raised by third parties that portions of Figure 1 are identical to Figure 1 in another article (1) from a different author group and covering a different research topic. Further investigation confirmed these concerns and revealed additional duplication in Figures 1 and 3, overlapping with Figures 1 and 2 in (1). The authors of both articles were approached for an explanation but were unable to address the concerns raised. The authors of (1) did not respond, and the authors of this article were unable to provide any underlying data. The retraction has been agreed because of concerns regarding the integrity of the research presented given that the figures were duplicated.

(1) Zhang J, Wang R, Cheng L, Xu H. Celastrol inhibit the proliferation, invasion and migration of human cervical HeLa cancer cells through down‐regulation of MMP‐2 and MMP‐9. *J Cell Mol Med*. 2021; 25: 5335‐5338. https://doi.org/10.1111/jcmm.16488

